# eNOS expression and NO release during hypoxia is inhibited by miR-200b in human endothelial cells

**DOI:** 10.1007/s10456-018-9620-y

**Published:** 2018-05-08

**Authors:** Anna Janaszak-Jasiecka, Anna Siekierzycka, Sylwia Bartoszewska, Marcin Serocki, Lawrence W. Dobrucki, James F. Collawn, Leszek Kalinowski, Rafal Bartoszewski

**Affiliations:** 1 Department of Biology and Pharmaceutical Botany, Medical University of Gdansk, Hallera 107, 80-416 Gdańsk, Poland; 2 Department of Medical Laboratory Diagnostics and Central Bank of Frozen Tissues & Genetic Specimens, Medical University of Gdansk, Gdańsk, Poland; 3 Department of Inorganic Chemistry, Medical University of Gdansk, Gdańsk, Poland; 4 Department of Bioengineering, University of Illinois at Urbana-Champaign, Urbana, IL USA; 5 Beckman Institute for Advanced Science and Technology, Urbana, IL USA; 6 Biobanking and Biomolecular Resources Research Infrastructure Poland (BBMRI.PL), Gdańsk, Poland; 7 Department of Cell, Developmental and Integrative Biology, University of Alabama at Birmingham, Birmingham, USA

**Keywords:** eNOS, NOS3, MicroRNA 200b, Hsa-miR-200b-3p, Hypoxia, Nitric oxide bioavailability, Hypoxia-related diseases

## Abstract

The nitric oxide (NO) secreted by vascular endothelium is required for the maintenance of cardiovascular homeostasis. Diminished release of NO generated by endothelial NO synthase contributes to endothelial dysfunction. Hypoxia and ischemia reduce endothelial eNOS expression via posttranscriptional mechanisms that result in *NOS3* transcript destabilization. Here, we examine whether microRNAs contribute to this mechanism. We followed the kinetics of hypoxia-induced changes in *NOS3* mRNA and eNOS protein levels in primary human umbilical vein endothelial cells (HUVECs). Utilizing in silico predictive protocols to identify potential miRNAs that regulate eNOS expression, we identified miR-200b as a candidate. We established the functional miR-200b target sequence within the *NOS3* 3′UTR, and demonstrated that manipulation of the miRNA levels during hypoxia using miR-200b mimics and antagomirs regulates eNOS levels, and established that miR-200b physiologically limits eNOS expression during hypoxia. Furthermore, we demonstrated that the specific ablation of the hypoxic induction of miR-200b in HUVECs restored eNOS-driven hypoxic NO release to the normoxic levels. To determine whether miR-200b might be the only miRNA that had this effect, we utilized Next Generation Sequencing (NGS) to follow hypoxia-induced changes in the miRNA levels in HUVECS and found 83 novel hypoxamiRs, with two candidate miRNAs besides miR-200b that could potentially influence eNOS levels. Taken together, the data establish miR-200b-eNOS regulation as a first hypoxamiR-based mechanism that limits NO bioavailability during hypoxia in endothelial cells, and show that hypoxamiRs could become useful therapeutic targets for cardiovascular diseases and other hypoxic-related diseases including various types of cancer.

**Electronic supplementary material:**

The online version of this article (10.1007/s10456-018-9620-y) contains supplementary material, which is available to authorized users.

## Introduction

The vascular endothelium synthesizes and secrets a broad spectrum of substances including nitric oxide (NO) for the regulation of vascular tone and structure. This mediator is generated by endothelial NO synthase (eNOS encoded by *NOS3* gene) in endothelial cells (ECs) and diffuses to the surrounding tissues in order to relax smooth muscle cells [1]. NO also prevents leukocyte adhesion and migration into the arterial wall, muscle cell proliferation, platelet adhesion and aggregation, and adhesion molecule expression [1]. Hence, maintaining the proper NO levels is cardioprotective.

Impaired eNOS activity contributes to endothelial dysfunction and is involved in the pathomechanisms of several cardiovascular diseases including atherosclerosis and hypertension [1, 2]. Although calcium/calmodulin binding and phosphorylation by serine/threonine-specific kinase (Akt) [3] are the major mechanisms governing eNOS activity, numerous pathological factors including hypoxia/ischemia modulate eNOS expression at both the transcriptional [4, 5] and posttranscriptional levels [5–7]. Although during the early stages of hypoxia, eNOS levels were shown to be induced transcriptionally due to hypoxia-inducible factor (HIF) activity [8, 9], numerous reports have linked the loss of NO bioavailability under prolonged hypoxia and ischemia to the reduction of endothelial eNOS levels [10, 11].

To date, an eNOS-specific posttranscriptional mechanism was proposed to be responsible for the hypoxic decline in the *NOS3* mRNA half-life [12]. Under normoxic conditions, *NOS3* mRNA is stabilized by heterogeneous nuclear ribonucleoprotein E1 (hnRNPE1) complex, while during hypoxia this interaction is lost, making the *NOS3* transcript prone to destabilization by its natural antisense *sONE* RNA (also known as *ATG9B, NOS3AS*, and *APG9L2*). *sONE* binds to both the *NOS3* coding sequence and the 3′UTR [11–13]. Interestingly, however, impairing the microRNA function via Dicer silencing resulted in eNOS upregulation during normoxia, and thus suggested that these non-coding RNAs (ncRNAs) can provide a general posttranscriptional epigenetic mechanism for the regulation of eNOS expression [14]. Indeed, miR-155 [15], miR-214 [16], and miR-24 [17] were reported to directly bind to *NOS3* mRNA during normoxia, and a miR-200 family member, miR-200c, was shown to destabilize *NOS3* transcript in response to oxidative stress [18].

Impairment of hnRNPE1 binding to *NOS3* mRNA results in an increased susceptibility to downregulation by miR-765 in normoxic human umbilical vein endothelial cells (HUVECs) [12]; however, during hypoxia, access to this miRNA target site (TS) is impeded by *sONE*, and the physiological relevance of this interaction requires further clarification. Nevertheless, since *sONE* antisense only partially covers *NOS3* 3′UTR sequence [11], the hypoxic hnRNPE1 dissociation could reveal other miRNAs TSs at this 3′UTR, thus making this transcript sensitive for miRNA-based destabilization. Furthermore, hypoxia has been shown to specifically affect expression profiles of a large number of miRNAs [19].

Hence, it is very plausible that during hypoxia, specific miRNAs could directly reduce eNOS levels and consequently take a role in the physiological modulation of the bioavailability of NO [12], however, no such miRNA has been identified to date. In the present study, we identified a miR-200 family member, miR-200b, as a novel direct negative regulator of eNOS expression in human ECs in response to hypoxia. We also show that the specific ablation of hypoxic induction of miR-200b in HUVECs results not only in *NOS3* mRNA and eNOS protein rescue, but also in an increase in hypoxic NO release in situ.

## Methods

### Cell culture

Primary human umbilical vein endothelial cells pooled from ten individual donors(HUVECs) were purchased from Cellworks (Caltag Medsystems Ltd, UK) and cultured in EGM-2 Bulletkit Medium (Lonza). All experiments were conducted between passage 2 and 6 at a confluence of 70–80%. Human embryonic kidney 293 cells (HEK-293) were obtained from ATCC (CRL-1573) and cultured in Minimal Essential Media (MEM) supplemented with 10% Fetal Bovine Serum (FBS), 2 mM l-glutamine (Sigma-Aldrich), and antibiotics (100 units/mL of penicillin, 100 µg/mL of streptomycin; Sigma-Aldrich).

### Induction of hypoxia

Hypoxia was induced in a CO_2_/O_2_ incubator/chamber for hypoxia research (Invivo2 Baker Ruskin). Briefly, cells were cultured in 35 mm dishes at 0.9% O_2_ for the time periods specified. Control cells were maintained in normoxia in a CO_2_/O_2_ incubator (Binder).

### miRNA analog transfections

Cells were seeded onto 6-well plates or 35 mm dishes and transfected at 70–80% confluence with Lipofectamine RNAiMax (Thermo Fisher Scientific) according to the manufacturer’s protocol. mirVana miRNA mimics and mirVana miRNA Inhibitors (Thermo Fisher Scientific) were used at final concentrations of 20 and 50 nM, respectively. mirVana mimics and inhibitors used in this study: miR-200b mimic (Assay ID: MC10492) and inhibitor (Assay ID: MH10492). In all experiments, cel-miR-67 was used as a scramble control since it has no homology to any known mammalian miRNA (Assay ID: MC22484) [20]. The degree of miRNA overexpression or knockdown was determined by qRT-PCR. Following the transfection, cells were cultured for 48 h prior to analysis.

### Isolation of RNA and microRNA

Total RNA containing the microRNA fraction was isolated using miRNeasy kit (Qiagen). RNA concentrations were calculated based on the absorbance at 260 nm. RNA samples were stored at -70 °C until use.

### Measurement of mRNA and miRNA levels using quantitative real-time PCR (qRT-PCR)

We used TaqMan One-Step RT-PCR Master Mix Reagents (Applied Biosystems) as described previously [21, 22] using the manufacturer’s protocol. The relative expressions were calculated using the ${2^{ - \Delta \Delta {C_{\text{t}}}}}$ method [23], with TATA-binding protein (*TBP*) and *RNU44* genes as reference genes for the mRNA and miRNA, respectively. TaqMan probes ids used were *NOS3* (Assay ID: Hs00176166_m1); *TBP* (Hs00427620_m1); hsa-miR-200b (Assay ID: 002251); hsa-miR-200c (Assay ID: 002300); hsa-miR-429 (Assay ID: 001024); hsa-miR-210 (Assay ID: 000512) and *RNU44* (Assay ID: 001094).

### Western Blots

Cells were lysed in RIPA buffer (150 mM NaCl, 1% NP-40, 0.5% sodium deoxycholate, 0.1% SDS, 50 mM Tris–HCl, pH 8.0) supplemented with protease inhibitor [Complete Mini (Roche)] on ice for 15 min. The insoluble material was removed by centrifugation at 15,000×*g* for 15 min. Protein concentrations were determined by BioRad™ Protein Assay using bovine serum albumin (BSA) as a standard. Following the normalization of protein concentrations, lysates were mixed with an equal volume of 2× Laemmli sample buffer and incubated for 5 min at 95 °C prior to separation by SDS PAGE on stain-free TGX gradient gels (BioRad). Following SDS–PAGE, the proteins were transferred to polyvinylidene fluoride membranes (300 mA for 180 min at 4 °C). The membranes were then blocked with BSA (Sigma-Aldrich) dissolved in PBS/Tween-20 (3% BSA, 0.5% Tween-20 for 1–2 h), followed by immunoblotting with the primary antibody: anti-eNOS (1:1500, 610,297; BD Biosciences); anti-β-Actin (1:1000, ab1801; Abcam). After the washing steps, the membranes were incubated with goat anti-rabbit IgG (H+L chains) or with goat anti-mouse IgG (H+L) HRP-conjugated secondary antibodies (BioRad) and detected using ECL (Amresco). Densitometry was performed using Image Lab software v. 4.1 (BioRad).

### Luciferase reporter assays

A human 3′UTR *NOS3* firefly luciferase reporter construct (Vn) (HmiT088415-MT06) and its control vector (Vc) (CmiT000001-MT06) were purchased from GeneCopoeia. The reporter vector bearing the mutated *NOS3* 3′UTR (Vmut) was obtained by site-directed mutagenesis. Briefly, the template DNA (wild-type (Vn) vector) was amplified with mutation-introducing primers: Fwd: GTCTAATCTCTAAATCAgtcgacTATTATTGAAGATTTACC and Rev: GGTAAATCTTCAATAATAgtcgacTGATTTAGAGATTAGAC. The template DNA was then removed by digestion with *DpnI*, and the remaining mutated DNA was transformed into DH5α *E.coli* competent cells. Next, the designed Vmut vector was isolated from selected clones using GeneJET Plasmid Maxiprep Kit (Thermo Fisher Scientific). The correct mutagenesis clone was confirmed with Sanger sequencing. To test the posttranscriptional activity of the human *NOS3* 3′UTR regions, HEK293 cells were transfected with the constructs described above or with control plasmid. Twenty-four hours before the transfections, cells were seeded onto 24-well plates at ~80% confluency and then transfected using Lipofectamine 2000 (Thermo Fisher Scientific). Each well received 200 ng of total plasmid DNA and miR-200b/miR-200c mimics and or the cel-miR-67 scramble control at final concentration of 10 nM. 48 h after transfection, the cells were lysed using luciferase assay lysis buffer (Promega) and firefly/Renilla luciferase activities were measured using the Dual-Luciferase Reporter Assay (Promega) according to the manufacturer’s protocol. Results were plotted as a relative decrease in arbitrary light units compared with control cells.

### NO measurements in cell culture

The concentration of NO was measured with an electrochemical microbiosensor. The microsensor was prepared as described previously [24]. To analyze the NO levels, the three-electrode system was used that consists of the sensor-working electrode, a platinum wire (0.1 mm) counter electrode, and a standard calomel reference electrode. The NO concentration was proportional to the current measured by a porphyrinic sensor in amperometric mode (EG&G PAR model 283 Potentiostat/Galvanostat) at constant potential of 0.65 V. A linear calibration curve was constructed from 500 nmol/L to 5 µmol/L before the measurements with aliquots of the NO standard solutions. eNOS was stimulated with 1 µmol/L of calcium ionophore (CaI; A23187). NO was measured as an increase of the current from its background level in the presence or absence of 300 µmol/L L-NAME (eNOS inhibitor) [24–26].

### Next generation sequencing analyses of miRNAs

HUVECs at passage 3 were incubated under normoxic or hypoxic conditions for 16 h, and subsequently used for the RNA isolation and analyses. The induction of hypoxia was verified by accessing miR-210 levels prior to further analysis. MiRNA sequencing libraries were prepared using QIAseq miRNA library kit (Qiagen) following the manufacturer’s instructions and followed by sequencing on an Illumina NextSeq instrument. Using Qiagen’s Gene Globe Software, sequencing reads were aligned to the human reference genome assembly (hg19) followed by transcript assembly and estimation of the relative abundances. The analyses of the differential expression of small RNAs between the control (normoxia) and the experimental sample (16 h hypoxia) were performed with geNorm [27] the Gene Globe Software. Two independent pools of primary HUVECs were used in Next Generation Sequencing Experiments from two different laboratories: the UAB Heflin Center at UAB and the Medical University of Gdansk, Poland). The significant changes in miRNA levels (twofold changes), *p* value < 0.005) were only considered significant if they were seen in both experiments.

#### Statistical analysis

Results were expressed as mean ± standard deviation. Statistical significance was determined using the Student’s *t* test, with *p* < 0.05 considered significant. The correlation was accessed via Pearson product-moment correlation method.

## Results

### The hypoxic reduction in eNOS levels negatively correlates with miR-200b expression

Although previous studies have reported that eNOS protein and mRNA levels are reduced during hypoxia in human ECs [11, 13, 28], our goal was to understand the kinetics of hypoxia-induced changes in eNOS levels. To accomplish this, we performed time-course studies during hypoxia in HUVECs.

**Fig. 1:**
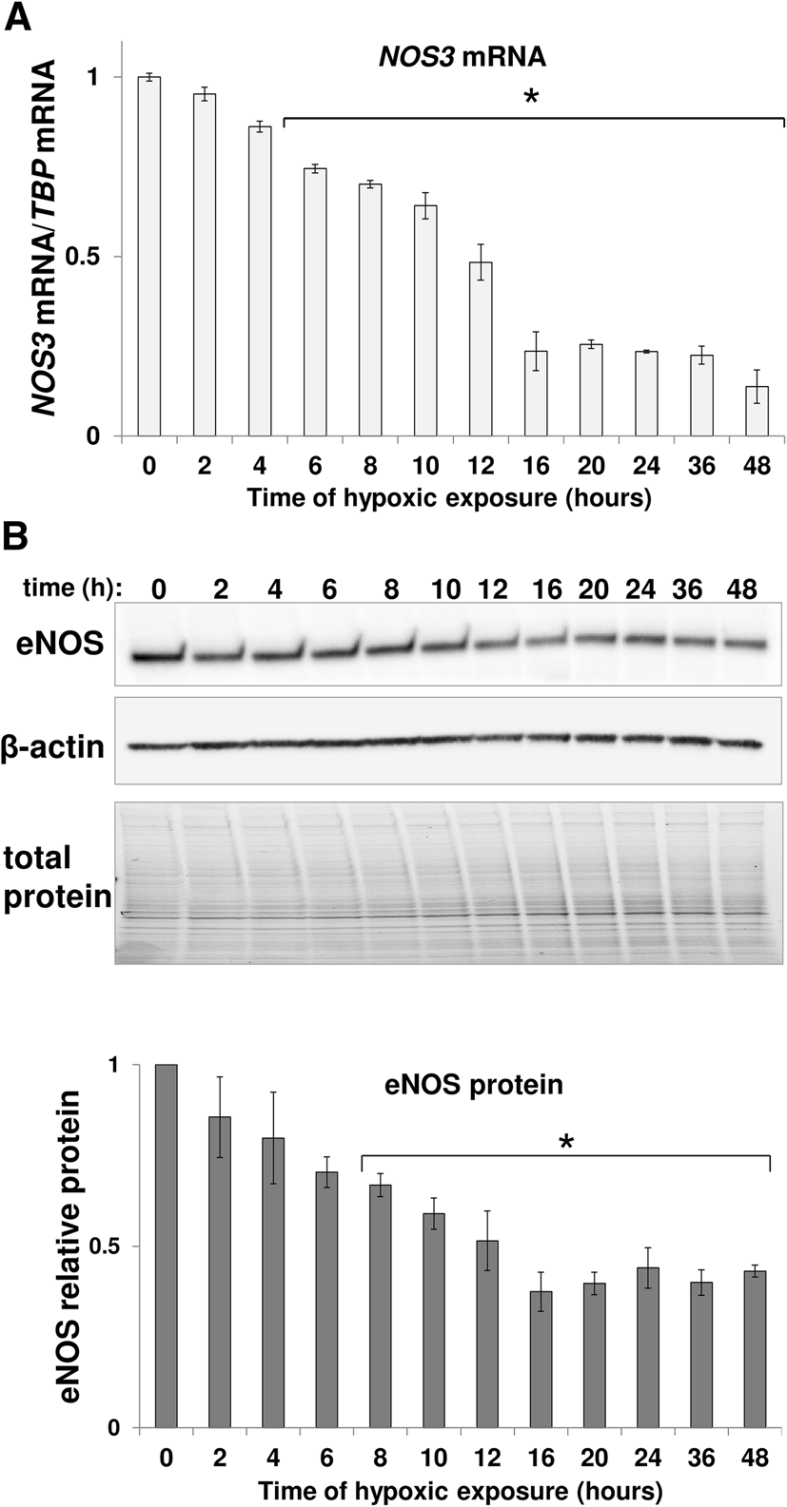
Hypoxia reduces eNOS expression in HUVECs. HUVECs were exposed to hypoxia for the time periods specified and total RNA and protein lysates were collected. **a** *NOS3* mRNA levels were quantified by qRT-PCR and normalized to *TBP* mRNA level. Data represent the mean ± SD of two independent experiments. **b** The corresponding changes in eNOS protein levels were evaluated by western blot normalized to β-Actin and total protein levels and related to normoxic control. *p < 0.05 was considered significant

This analysis indicated that *NOS3* mRNA is gradually reduced in response to hypoxia, as shown in Fig. 1a, reaching about 50 and 20% of its initial level after 12 and 24 h, respectively. The changes in *NOS3* mRNA correlated well (correlation coefficient 0.965; *p* value 2.2 E-14) with the eNOS protein changes (Fig. 1b). During the early stages of hypoxia, the eNOS protein levels gradually decrease for the first 16 h, and then remain essentially unchanged up to 48 h (Fig. 1b).

**Fig. 2:**
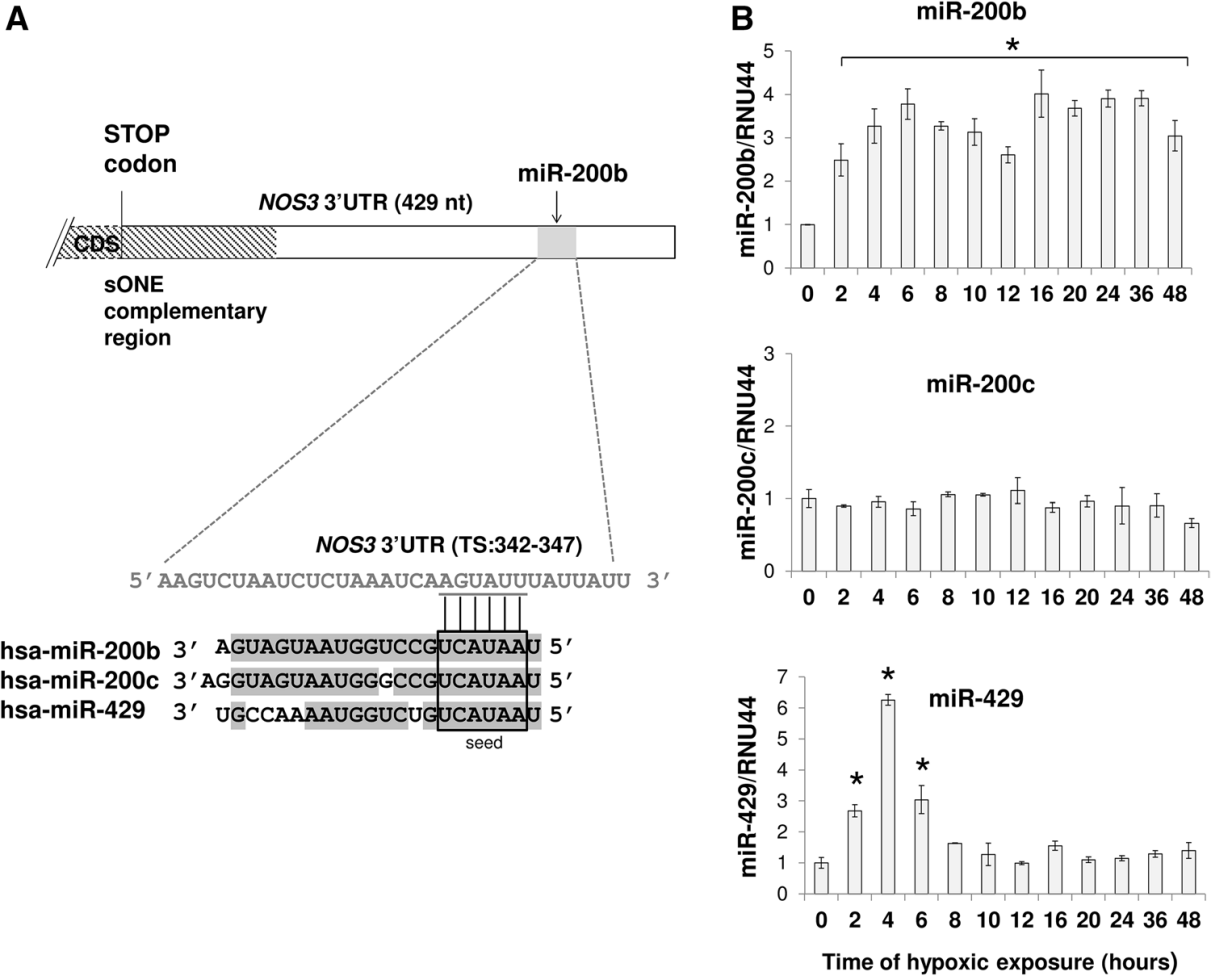
miR-200b and miR-429 are upregulated in response to hypoxia. **a** The schematic representation of the interaction of the miR-200b/200c/429 seed sequence with the predicted target sequence (TS) in the *NOS3* 3′UTR sequence. As indicated by shading, the miR-200b, miR-200c, and miR-429 share high sequence homology. **b** HUVECs were exposed to hypoxia for the times indicated and total RNA enriched in miRNAs was isolated. miR-200b, mir-200c, and miR-429 levels were quantified by qPCR and normalized to RNU44. Data represent the mean ± SD of three independent experiments. *p < 0.05 was considered significant

To test the hypothesis that hypoxia-induced miRNAs contribute to eNOS downregulation, we analyzed the *NOS3* 3′UTR sequence for potential hypoxamiR’s target sites using the miRANDA and TargetScan algorithms [29, 30]. Using this approach, we identified a potential 6mer target site for miR-429/200b/200c at position 342–347 from the stop codon in the 3′UTR of *NOS3* mRNA (Fig. 2a).

Although the expression of these miRNAs was previously reported to be hypoxia-dependent in ECs [22, 31, 32], we verified their expression profiles during hypoxia in HUVECs. As shown in Fig. 2, miR-200b was induced up to fourfold and remained elevated throughout the 48-h test period, while miR-429 was induced only during acute hypoxia (2–6 h), and miR-200c was not elevated at all and therefore is not involved in eNOS regulation during hypoxia. More importantly, the miR-200b hypoxic expression profile correlated negatively with the corresponding changes in eNOS protein and *NOS3* mRNA (correlation coefficients − 0.743 and − 0.639; *p* values of 0.000032 and 0.00077 for protein and mRNA, respectively), whereas no similar correlation was observed for miR-429 and miR-200c (correlation coefficients 0.423 and 0.262, respectively; *p* values of 0.039 and 0.214, respectively). This analysis supports miR-200b’s role in regulating eNOS expression during hypoxia.

### miR-200b binds to the NOS3 3′UTR

**Fig. 3:**
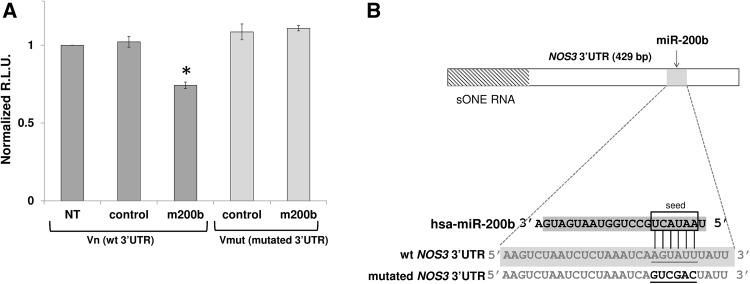
*NOS3* 3′UTR is a direct target for miR-200b, miR-200c, and miR-429. **a** Luciferase reporter constructs containing wild-type (Vn) or mutated (Vmut) *NOS3* 3′UTR sequence were transfected together with miR-200b or scramble control into HEK-293 cells. As a control reporter vector without 3′UTR (Vc) was used. The normalized R.L.U luciferase activities were calculated as a Vn/Vc or Vmut/Vc ratio (*n* = 2; *p < 0.05 vs. Vn/Vc). Data represent the mean ± SD of three experiments. *p < 0.05 was considered significant. **b** The schematic presentation of the mutations (bold) introduced into miR-200b TS in *NOS3* 3′UTR sequence

Although the miRNAs recognize specific target sequences, these sequences (6–8 nt) can be present in the 3′UTRs of many different genes. Hence, in order to exclude indirect effects of miR-200b on eNOS expression, we utilized a 3′UTR luciferase reporter. Briefly, a plasmid containing the 3′UTR of human *NOS3* gene was tested in a luciferase gene construct that was expressed in HEK293 in the presence and absence of a miR-200b analog (mimic). As shown in Fig. 3a, miR-200b overexpression resulted in significantly reduced luciferase expression compared to control. Furthermore, a similar experiment with reporter vector containing the 3′ UTR of human *NOS3* gene with a mutated miR-200b target site (Fig. 3b) did not result in a luciferase signal reduction (Fig. 3a), confirming a direct interaction between miR-200b and its target site at 3′UTR of *NOS3* mRNA.

**Fig. 4:**
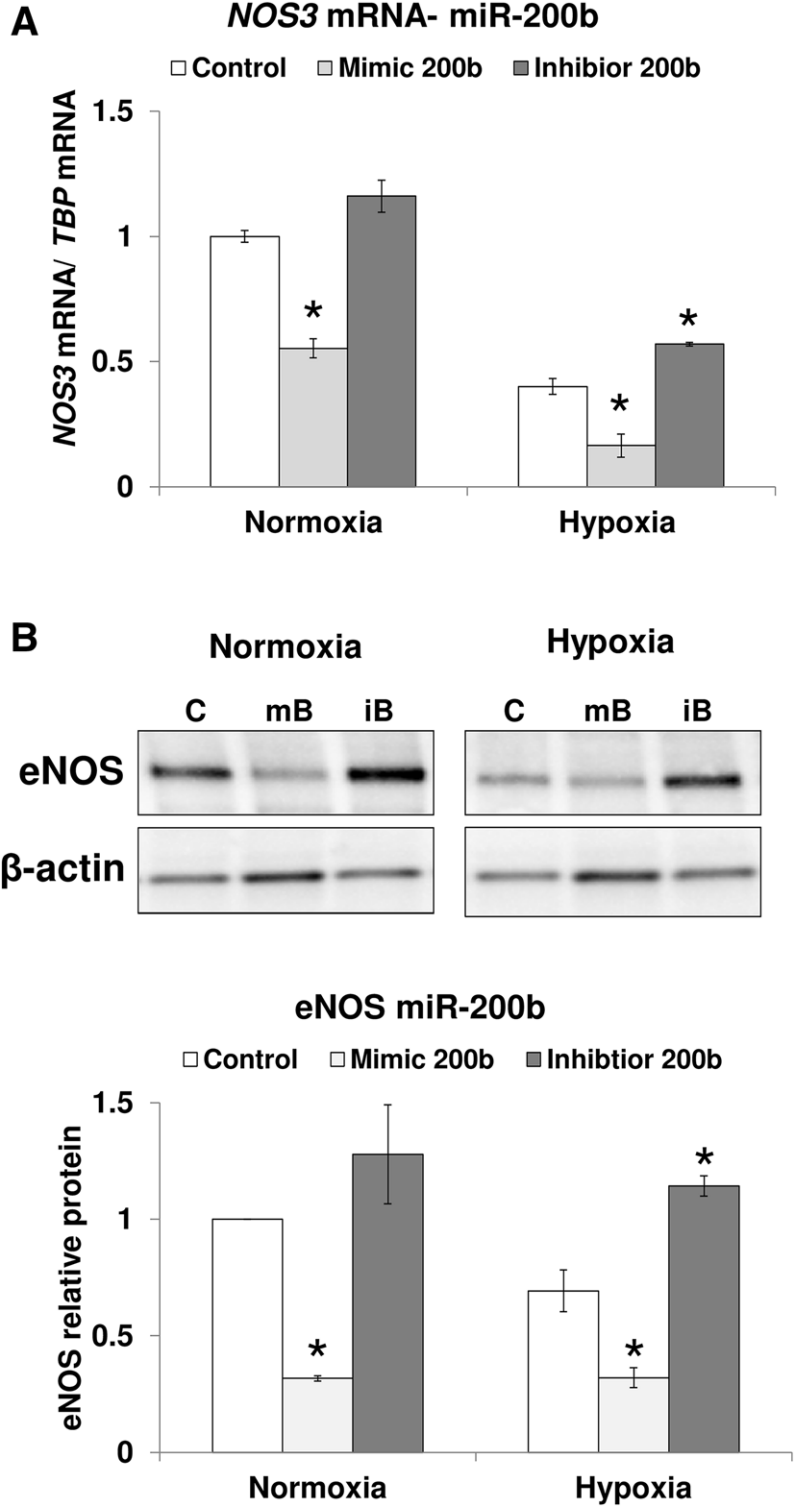
miR-200b downregulates eNOS expression in response to hypoxia. HUVECs were transfected with miR-200b mimic, mir-200b antagomiR, or scramble control and cultured in normoxia or hypoxia for 12 h. **a** *NOS3* mRNA levels were quantified by qRT-PCR and normalized to *TBP* mRNA. Data represent the mean ± SD of two independent experiments. **b** Corresponding changes in eNOS protein levels were monitored with western blot and normalized to β-Actin and related to the normoxic control. Data represent the mean ± SE of two independent experiments. *p < 0.05 was considered significant

Next, we tested the effects of miR-200b overexpression and inhibition on *NOS3* mRNA levels after 12 h of hypoxia in HUVECs. The miR-200b upregulation with mimic reduced *NOS3* mRNA in both hypoxia and normoxia (Fig. 4a). The inhibition of miR-200b activity with antagomiR increased *NOS3* mRNA significantly only during hypoxia (Fig. 4a), supporting the requirement of hnRNPE1 dissociation from *NOS3* mRNA for the miR-200b-*NOS3* functional interaction to occur. In parallel, we followed miR-200b analog effects on eNOS protein levels. As shown in Fig. 4b, both in normoxia and during hypoxia, miR-200b overexpression resulted in the reduction of eNOS protein levels in HUVECs. Notably, the antagomiR treatment during hypoxia had elevated eNOS protein, confirming the physiological effect of miR-200b on *NOS3* expression during low oxygen levels (Fig. 4b). Importantly, despite the rather small miR-200b antagomiR effect on *NOS3* mRNA during hypoxia, the attenuation of miR-200b expression restored eNOS protein to normoxic levels. This suggests that miR-200b effects on hypoxic eNOS expression are mainly caused by miRNA-mediated translational inhibition presumably due to low *NOS3* mRNA stability during hypoxia.

### Hypoxia miR-200b induction attenuates NO bioavailability

**Fig. 5:**
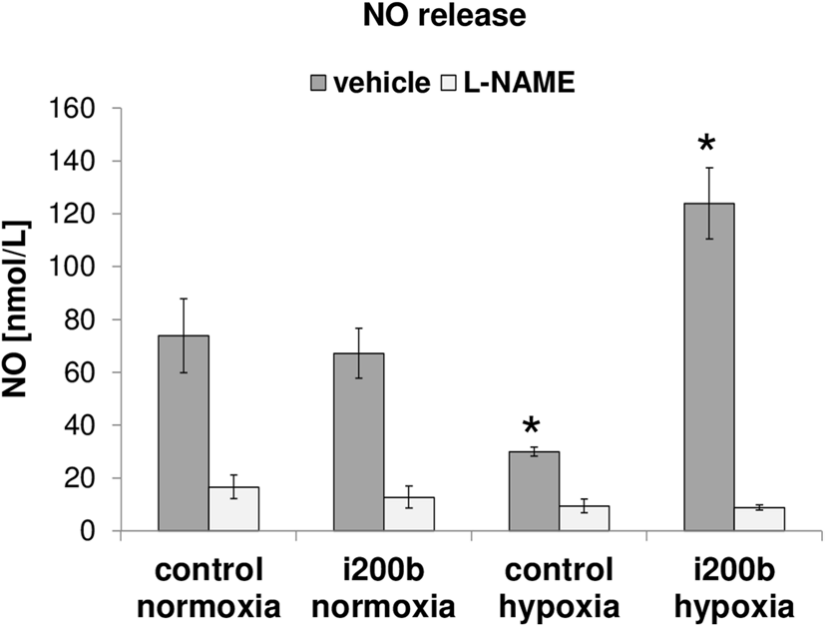
Hypoxia-inducible miR-200b impairs NO release. HUVECs were transfected with miR-200b antagomiR or scramble control, the cells were then cultured in normoxia or hypoxia conditions for 16 h, and the NO release was measured. eNOS was stimulated by 1.0 µmol/L calcium ionophore. The specific eNOS inhibitor NG-nitro-l-arginine methyl ester (L-NAME) was used as a control. Results represent the mean ± SD of three measurements, *p < 0.05 was considered significant

Given the effects on the eNOS RNA and protein, we next tested the effect of miR-200b antagomiR on NO release. The direct measurements of NO released from HUVECs cultured during hypoxia for 16 h indicate that eNOS produces approximately 50% less NO during hypoxia compared to normoxia (Fig. 5), confirming previous reports [33]. Furthermore, decreasing the physiological miR-200b levels with antagomiR had little effect on NO release during normoxia, but had a dramatic fourfold increase in eNOS-specific NO release (Fig. 5**)**. This confirmed miR-200b as an important regulator limiting NO bioavailability during hypoxia. Furthermore, the antagomiR had no effect on NO release from normoxic cells, supporting the role of hnRNPE1 in stabilizing *NOS3* transcript during normoxia. As expected, the release of NO after eNOS stimulation was dramatically inhibited by 300 μmol/L L-NAME in all sets of experiments.

## Discussion

The mediator generated by eNOS, NO, is a key component of the vascular homeostasis [25, 34–37] and the loss of NO bioavailability provides an independent risk factor for cardiovascular diseases [35, 36]. Many of these disorders are associated with hypoxia or ischemia in different organs, while hypoxia and ischemia lower endothelial eNOS expression and lead to loss of NO bioavailability [10, 11]. Therefore, understanding the cellular pathways that regulate eNOS expression and consequently NO release during hypoxia is important.

The hypoxic loss of NO release is mediated by decreased eNOS activity brought about by altered eNOS phosphorylation [33], whereas posttranscriptional mechanisms that reduce the stability of *NOS3* mRNA have been proposed to be another major contributing factor in the loss of NO bioavailability. The hnRNPE1 complex stabilizes *NOS3* mRNA during normoxia by preventing binding factors that negatively affect the transcript half-life [12]. During hypoxia, however, hnRNPE1 protection is missing and this makes the *NOS3* transcript accessible to destabilizing factors such as *sONE* [11–13]. However, attenuation of *sONE* in hypoxic ECs only partially restores *NOS3* expression [11], and thus involvement of other posttranscriptional mechanisms must contribute to the observed reduction in the *NOS3* mRNA levels during hypoxia.

Although the role of miRNAs in posttranscriptional gene regulation is clearly established, it is now evident that recent specific alterations in miRNA expression can also regulate eNOS levels [14–18] (reviewed in [6]). Moreover, differences in miRNA expression are also present in cardiovascular disorders [38], suggesting that miRNA network changes could influence disease pathogenesis. Despite the fact that inhibiting miRNAs function (through DICER silencing [12]) prevents eNOS downregulation, only five miRNAs that directly affect *NOS3* expression have been identified that include miR-214 [16], miR-155 [15], miR-24 [39], miR-765 [12], and miR-200c [18]. However, previous studies did not examine whether miRNA-based mechanisms contribute to eNOS levels reduction during hypoxia.

**Fig. 6:**
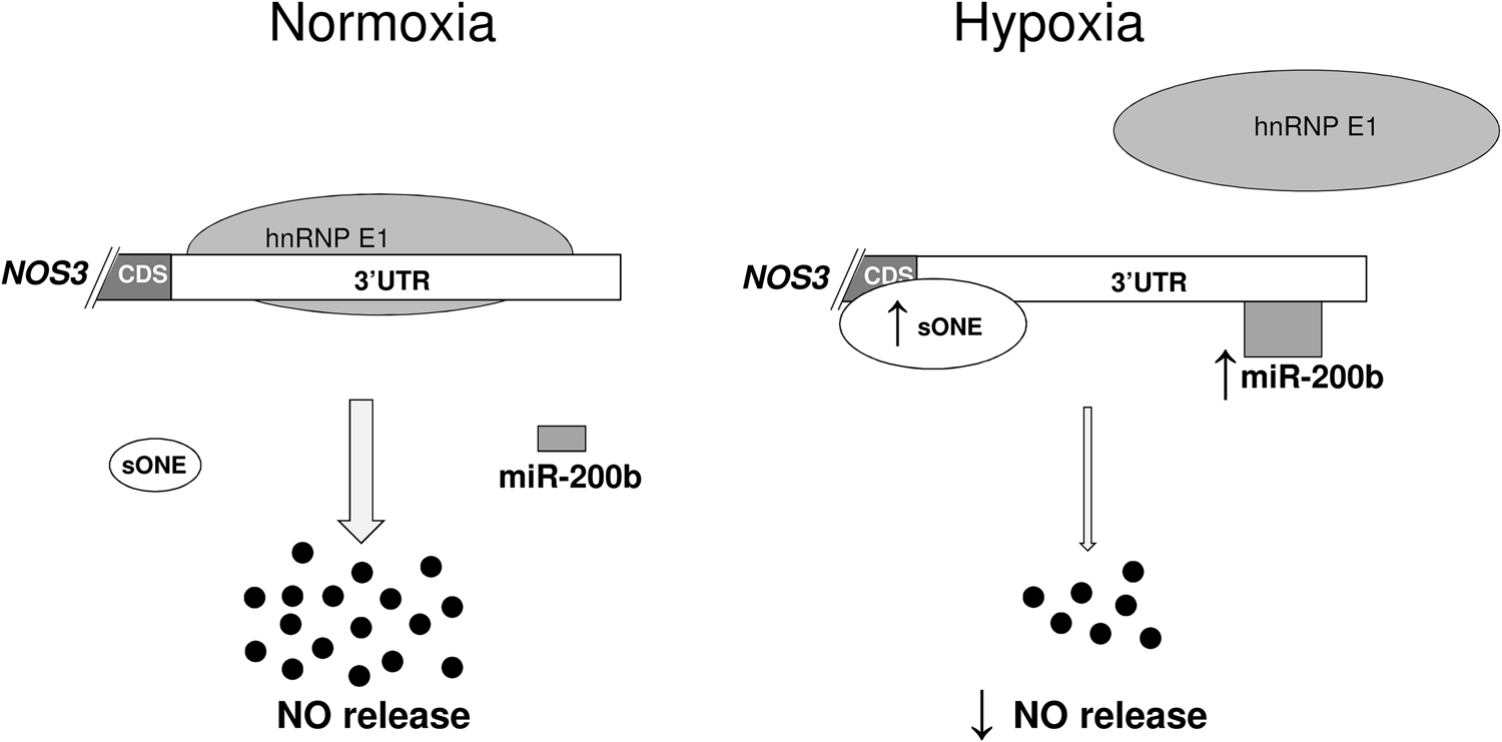
Hypoxia-inducible miR-200b reduces NO release in ECs by direct downregulation of eNOS expression. In normoxic ECs, *NOS3* mRNA is actively stabilized by hnRNP E1 containing complex. Under hypoxia, hnRNP E1 dissociates from *NOS3* 3′UTR making it prone to destabilization by hypoxia-induced: miR-200b and *sONE* RNA. The hypoxic destabilization of *NOS3* mRNA by miR-200b results in reduced eNOS protein and diminished NO release

hnRPE1 dissociates and destabilizes *NOS3* mRNA during hypoxia through *sONE* association (Fig. 6). It is also plausible that binding of *sONE* to *NOS3* 3′UTR would prevent miRNAs binding to this sequence. However, impairing *sONE* expression during hypoxic only partially attenuated the fall in both *NOS3* mRNA and eNOS protein (~ one-half fold), whereas hypoxia results in a much more pronounced eNOS downregulation (~ fivefold drop) [11]. Notably, however, *sONE* occupies only one-third of *NOS3* 3′UTR (Fig. 2a), whereas the remaining sequence, due to hnRNPE1 dissociation, becomes available for miRNAs binding.

This hypothesis is supported by a previous study in which siRNA-mediated impairment of hnRNP1 sensitized *NOS3* transcript to destabilization by miR-765 in normoxic HUVECs [12]. However, miR-765 levels are reduced during hypoxia [40], and whether the hnRNPE1-dependent mechanism of *NOS3* mRNA destabilization requires changes in miRNA levels is unclear. In contrast, numerous miRNAs are induced during hypoxia in ECs and the hnRNPE1-dependent assessment to these miRNA target sites at *NOS3* 3′UTR would allow dynamic and bidirectional modulation of *NOS3* mRNA stability. Taken together, this suggests that hypoxamiRs contribute to the decline in *NOS3* message and protein expression, and importantly provide intriguing targets for elevating NO levels during hypoxia.

Our in silico predictions indicate that miR-200b, miR-200c, and miR-429 were putative candidates for eNOS posttranscriptional regulation during hypoxia. Furthermore, the predicted target site for these miRNAs was shown to be target of miR-200c under oxidative stress [18]. To differentiate between miR-200b and miR-200c and miR-429, we utilized a hypoxia time course in HUVECs, and demonstrate a correlation between miR-200b induction and a corresponding decline in *NOS3* mRNA and protein levels, whereas miR-200c and miR-429 changes did not correlate. Using the 3′UTR *NOS3* luciferase constructs, we also demonstrated that miR-200b had a direct effect on luciferase expression, and this clearly established a direct effect on *NOS3* message levels. Final support for the role of miR-200b comes from the negative and positive effects of miR-200b mimics and antagomirs on eNOS expression. Importantly, the miR-200b inhibition significantly increased eNOS levels under hypoxia exclusively, confirming that this miRNA regulates eNOS expression via an hnRNPE1-dependent mechanism. Taken together, the results suggest that during low oxygen conditions which could occur in various cardiovascular pathologies, miR-200b is upregulated and has a direct inhibitory effect on *NOS3* message and eNOS protein expression in human ECs.

The removal of physiological miR-200b levels with antagomiR in hypoxic HUVECs resulted in the restoration of eNOS which released NO above normoxic levels and about fourfold above the hypoxic ones. This result shows that the hypoxic induction of miR-200b not only leads to reduced eNOS levels but also to reduced NO release as well. This was demonstrated in the most accurate method by using the NO-detecting porphyrinic biosensor, designed for cell cultures, that allows direct quantification of biologically active NO with high sensitivity in situ and in real time. This methodological approach for NO measurements provides the evidence on whether the changes in eNOS expression in endothelial cells can be truly translated into the increase of NO bioavailability [24–26].

This main finding of our study highlights the role of posttranscriptional mechanisms modulating eNOS expression for NO bioavailability. However, by inhibiting miR-200b function during hypoxia, we not only restored NO production, but we also increased it above normoxic levels. This can be explained by the network of miR-200 family targets involved in hypoxic response [22, 31, 32] that could mediate other mechanisms indirectly affecting eNOS activity during hypoxia.

The approach that we used for examining the miR-200b-eNOS interaction, however, does not answer the question about the potential role of other hypoxiamiRs in this process. To address this, we utilized Next Generation Sequencing (NGS) to follow hypoxia-induced changes in the miRNA levels in HUVECS. After 16 h of hypoxia, we observed a significant reduction of *NOS3* mRNA and interestingly, 72 miRs were significantly induced, while 15 were reduced (Table 1). Among these 72 miRNAs, only 4 had been previously reported as hypoxiamiRs (including miR-210). Deregulation of many others, however, has been associated with various types of cancer (Table 1). In order to predict which miRNAs might govern hypoxic eNOS expression, we utilized the miRDIP database. This database effectively combines different target prediction algorithms in order to identify the most probable interactions [41]. The highest scores and thus the highest probability hits for binding to the *NOS3* 3′UTR were miR-424-5p and miR-503-5p. Their miRDIP scores were 0.322 and 0.265, respectively, and as a comparison, the score for miR-200b using this analysis was 0.212. Furthermore, both of these miRNAs were previously reported in ECs as hypoxiamiRs [42–44]. Whether or not they influence *NOS3* mRNA expression levels, however, they will require experimental validation. Taken together, our deep sequencing analysis of the hypoxia effect on miRNAs expression in human primary ECs resulted in identification of 83 novel hypoxiamiRs. Among this group, 30 have previously been shown to be dysregulated in cancer cells.

**Table 1:** Hypoxia-induced changes in miRNAs levels in HUVECs. Cells were exposed to hypoxia for 16 h and miRNA profiles determined with NGS analysis of two independent experiments

MiRNA induced in HUVECs during 16 h of hypoxia				
miRNA	Fold change	*NOS3* target site _(mirDIP integrated score)_	Process affected	References
hsa-miR-210-3p	31.07	0.115	HypoxiamiR (ECs and many other types of cells)	[45–47]
hsa-miR-1279	21.07	0.050	–	–
hsa-miR-4256	18.52	0.008	–	–
hsa-miR-4700-3p	16.74	0.052	–	–
hsa-miR-3131	14.72	0.011	Dysregulated in gastric cancer	[48]
hsa-miR-33a-5p	10.47	0.026	Dysregulated in melanoma and hepatocellular carcinoma	[49, 50]
hsa-miR-4680-3p	10.25	0.035	Dysregulated in glioma	[51]
hsa-miR-6744-3p	9.01	0.014	–	–
hsa-miR-6789-5p	6.95	0.011	–	–
hsa-miR-4771	6.91	0.034	–	–
hsa-miR-4710	6.64	0.010	Upregulated in HUVECs exposed to estradiol	[52]
hsa-miR-3168	6.56	0.048	–	–
hsa-miR-6828-3p	6.31	0.014	–	–
hsa-miR-4303	5.70	0.046	–	–
hsa-miR-4433a-3p	5.55	0.095	–	–
hsa-miR-6820-3p	5.48	0.014	–	–
hsa-miR-6502-5p	5.32	0.014	–	–
hsa-miR-5095	5.19	0.070	–	–
hsa-miR-6850-3p	4.77	0.012	–	–
hsa-miR-105-5p	4.65	0.044	Dysregulated in breast cancer, hepatocellular carcinoma, and glioma	[53–55]
hsa-miR-4659a-5p	4.61	0.014	–	–
hsa-miR-4468	4.54	0.067	–	–
hsa-miR-649	4.44	0.024	Dysregulated in bladder cancer	[56]
hsa-miR-200b-5p	4.42	0.012	Dysregulated in breast, head, and neck cancers	[57, 58]
hsa-miR-944	4.37	0.034	Dysregulated in breast, gastric, and colorectal cancers	[59–61]
hsa-miR-6514-5p	4.30	0.013	–	–
hsa-miR-4322	4.25	0.012	–	–
hsa-miR-8052	4.19	0.020	–	–
hsa-miR-4450	4.08	0.096	–	–
hsa-miR-206	4.02	0.086	HypoxamiR (rat PASMCs, and rat cardiomyocytes)	[62]
hsa-miR-6131	3.90	0.066	–	–
hsa-miR-5189-3p	3.88	0.013	–	–
hsa-miR-639	3.79	0.088	Dysregulated in breast and tongue cancers	[63, 64]
hsa-miR-3916	3.79	0.091	–	–
hsa-miR-4477a	3.72	0.015	–	–
hsa-miR-3679-3p	3.71	0.009	Dysregulated in non-small cell lung cancer	[65]
hsa-miR-503-3p	3.62	0.031	Dysregulated in breast cancer	[66]
hsa-miR-433-5p	3.45	0.013	–	–
hsa-miR-1266-5p	3.44	0.031	Dysregulated gastric in cancer	[67]
hsa-miR-6827-5p	3.37	0.012	–	–
hsa-miR-4285	3.35	0.012	–	–
hsa-miR-3606-5p	3.34	0.016	–	–
hsa-miR-5590-5p	3.31	0.016	–	–
hsa-miR-215-3p	3.28	0.015	–	–
hsa-miR-505-5p	3.27	0.013	Dysregulated in breast cancer	[68]
hsa-miR-562	3.25	0.044	Dysregulated in prostate cancer	[69]
hsa-miR-4687-5p	3.23	0.028	Dysregulated in colorectal cancer	[70]
hsa-miR-518d-3p	3.13	0.020	–	–
hsa-miR-5681a	3.13	0.033	–	–
hsa-miR-424-3p	3.07	0.034	Dysregulated colorectal in cancer	[71]
hsa-miR-4682	3.05	0.082	–	–
hsa-miR-4772-5p	3.05	0.013	–	–
hsa-miR-3117-3p	2.97	0.014	Dysregulated in hepatocellular carcinoma	[72, 73]
hsa-miR-7162-3p	2.95	0.013	–	–
hsa-miR-891a-5p	2.90	0.048	–	–
hsa-miR-4725-3p	2.89	0.012	–	–
hsa-miR-617	2.84	0.044	–	–
hsa-miR-3621	2.84	0.036	Dysregulated in colorectal cancer	[74]
**hsa-miR-503-5p**	**2.71**	**0.266**	**HypoxiamiR (rat MSCs)**	[42]
hsa-miR-512-3p	2.62	0.039	Dysregulated in anaplastic large cell lymphoma and prostate cancer	[75, 76]
hsa-miR-144-3p	2.50	0.045	Dysregulated in papillary thyroid carcinoma and renal cell carcinoma	[77, 78]
hsa-miR-24-1-5p	2.49	0.009	Dysregulated in prostate cancer	[79]
hsa-miR-5007-3p	2.46	0.009	Dysregulated in gastric cancer	[80]
hsa-miR-8066	2.46	0.009	–	–
**hsa-miR-424-5p**	**2.42**	**0.323**	**Hypoxiamir (HUVECs and many other cell lines)**	[43, 44]
hsa-miR-548bb-5p	2.34	0.009	–	–
hsa-miR-510-3p	2.31	0.009	–	–
hsa-miR-4500	2.27	0.015	Dysregulated in colorectal cancer	[81]
hsa-miR-6751-5p	2.26	0.017	–	–
hsa-miR-4700-5p	2.25	0.175	–	–
hsa-miR-802	2.20	0.040	Dysregulated in squamous cell carcinoma	[82]
hsa-miR-4735-5p	2.18	0.009	–	–

MiRNA reduced in HUVECs during 16 h of hypoxia				
hsa-miR-483-3p	0.50	0.020	Dysregulated in squamous cell carcinoma	[83]
hsa-miR-6761-5p	0.45	0.041	–	–
hsa-miR-4668-5p	0.43	0.059	–	–
hsa-miR-106a-3p	0.42	0.035	Dysregulated in breast cancer	[84]
hsa-miR-4461	0.40	0.033	–	–
hsa-miR-6727-5p	0.30	0.009	–	–
hsa-miR-4472	0.28	0.031	Dysregulated in breast cancer	[85]
hsa-miR-7706	0.27	0.006	–	–
hsa-miR-922	0.25	0.163	Dysregulated in hepatocellular carcinoma	[86]
hsa-miR-4484	0.25	0.033	Dysregulated in glioblastoma	[87]
hsa-miR-6865-3p	0.25	0.014	–	–
hsa-miR-4483	0.20	0.067	–	–
hsa-miR-449c-5p	0.20	0.088	Dysregulated in nasopharyngeal carcinoma	[88]
hsa-miR-6785-5p	0.13	0.072	–	–
hsa-miR-599	0.12	0.020	Dysregulated in breast and non-small cell lung cancer	[89, 90]

The table represents significant changes (fold change 2, *p* value < 0.005), that were consistent between the two independent experiments. The raw data are provided in Supplemental file 1. Potential miRNA binding to *NOS3* 3′UTR was predicted with mirDIP, and miRs that resulted in the prediction of high score are marked bold. Previously reported hypoxiamiRs are underlined

In summary, the studies show that hypoxia-induced physiological changes in miR-200b levels in human ECs contribute directly to eNOS downregulation during hypoxia and lead to diminished NO release. Hence, these results complement previous studies indicating the hnRNPE1-dependent *sONE* direct posttranscriptional effects on the downregulation of eNOS during hypoxia with a mechanism that involves a hypoxia-induced miRNA (Fig. 6). Furthermore, stabilization of eNOS protein levels during hypoxia through inhibition of miR-200b’s effects and the corresponding increase in NO release may provide a novel therapeutic opportunity for increasing NO bioavailability during various cardiovascular diseases. Moreover, in human ECs exposed to hypoxia, using Next Generation Sequencing, we identified 83 novel hypoxamiRs and proposed that miR-424-5p and miR-503-5p are candidate miRNAs that could potentially regulate eNOS expression during hypoxia. Although our initial analysis focused on eNOS-miRNA interactions, we also identified a number of hypoxamiRs many of which may be important in the cancer hypoxic microenvironment [91, 92].

## Electronic supplementary material

Below is the link to the electronic supplementary material.

Supplementary material 1 (XLSX 704 KB)

Supplementary material 2 (XLSX 278 KB)

## Abbreviations

eNOS: Endothelial nitric oxide synthase
NO: Nitric oxide
HUVECs: Human umbilical vein endothelial cells
HIF: Hypoxia-inducible factor
